# Some Causes That Lead to Invalidism in Women

**Published:** 1894-12

**Authors:** J. B. S. Holmes

**Affiliations:** Atlanta, Ga.; Adjunct Professor of Obstetrics and Gynecology, Southern Medical College, Atlanta, Ga.; Vice-President of the Southern Surgical and Gynecological Association; President of the Tri-State Medical Association; Ex-President Georgia Medical Association; Member of the American Medical Association; Fellow of the American Association of Obstetricians and Gynecologists


### SOME CAUSES THAT LEAD TO INVALIDISM IN WOMEN.

BY J. B. S. HOLMES, M. D., ATLANTA, GA.,

Adjunct Professor of Obstetrics and Gynecology, Southern Medical College, Atlanta, Ga.; Vice-President of the Southern Surgical and Gynecological Association; President of the Tri-State Medical Association; Ex-President Georgia Medical Association; Member of the American Medical Association; Fellow of the American Association of Obstetricians and Gynecologists.

(CONCLUDED.)

Tait, in speaking of the over-taxed school-girl, says: "I desire to notice music especially, for I am quite certain the instruction in that art, as carried out in our boarding schools, has to answer for a great deal of menstrual mischief. To keep a young girl, during her first efforts of sexual development, seated upright upon a music stool, with her back unsupported, for several hours, can be nothing but mischief. This is most pernicious, and I have repeatedly had to trace to it the cause of serious disease in young ladies." The above is strong language coming from eminent authority. I think it would be best for the girl's musical education to commence only after the college curriculum has been completed, substituting in this curriculum, physical culture under the direction of well-educated teachers. If, after her college life is ended, she has a love and fondness for music, she then has ample time to take up the study, when her brain and nervous system will not be so taxed and she can pursue it without injury to herself. Twenty years of age is early enough for a girl to graduate. I must again beg your indulgence for one or two suggestions. There should be appointed in each State a Commission of Hygiene, composed of intelligent and educated physicians, selected by the Board of Censors of the State Medical Association and appointed by the Governor. In order to take it entirely out of politics, their appointment should be for life, removeable only for cause, especially incompetency. It should be the duty of this commission to visit the schools, particularly those for girls, at least once in each month, to look carefully into everything having a bearing on the physical condition of the girls, examine carefully into their menstrual condition, regulate the number of their studies and study hours, the amount and character of their exercise, etc. It would be well to have on this board a competent female physician to whom the school-girl would doubtless use more freedom of speech in regard to her physical disorders. The States could make, in my opinion, no expenditure so wise and one so likely to bring magnificent returns in changing the physical condition of our women from retrograde to advancement and ascendency.

Heredity undoubtedly plays a great part in furnishing us invalid women. Defects in the mother may, and are likely to be transmitted to the children. Especially is this true as regards malformations and malignant diseases. A daughter is much more prone to inherit a physically weak constitution from the mother than from the father. It is our duty to bring to bear every influence to prevent women with parents of tuberculous and cancerous history from marrying.

High-heeled shoes, especially when worn at an early age, when the articulations are not fixed, not only alter the shape of the foot, but are apt to cause a change in the inclination of the pelvis and the normal curvature of the spine. The body is placed in the same position as if standing on an inclined plane. This is not only fatiguing on the muscles of the leg and thigh, frequently producing neuralgia, but it is, in my opinion, often the cause of uterine displacement and spinal congestions, with its varied and disastrous reflex phenomena. While much has been said and written, pro and con, in regard to the corset, common sense will teach any thinking man, who has any knowledge of anatomy and physiology, that it is productive of a vast amount of injury to the woman. It must be remembered that the normal breathing of a woman, without a corset, is like that of a man,—abdominal. Change the type of this and we fail at once to get that free expansion of the lungs to take oxygen so necessary to the nutrition of every part of the body. The pelvic organs, in their unembarrassed state, move up and down with each respiration. This can be beautifully seen with no waist constriction during an examination of the uterus or vagina, or in doing plastic surgery on these organs. Any surgeon who has done this work will bear witness to the wonderful descent of the uterus from the pressure of the abdominal muscles in the act of vomiting. When the abdomen is pressed by a corset, sitting or bending forward greatly increases the internal abdominal pressure. By tight lacing the uterus is displaced downward, backward or forward and the pelvic circulation is affected, not only by this displacement, but most of the pressure of the corset is near the point where the ovarian veins empty and the pressure on these keeps up a constant pelvic hyperæmia. The abdominal muscles are thinned and weakened by pressure and chest expansion, by pressure on the lower ribs, is prevented. The movements of the diaphragm so necessary to abdominal breathing is in this way seriously interfered with. The action of the diaphragm produces the up and down movement of the pelvic organs—the alternating pressure and relaxation secures free circulation in the pelvic vessels. Stop these movements and produce compression of the vessels and you meet at once a pelvic disease, if not actually developed by this, the most favorable condition is produced from the most trivial cause, to set up active trouble there.

Another very frequent cause of disease in women is constipation. It is truly remarkable how careless many women are in this respect. The mother should educate the girl from infancy that it is just as important to keep the bowels open as to sleep and eat. We find girls frequently going from three to five days, in some instances much longer, without a movement from the bowels. Not only do they have from this a poisoning of the system from absorption of the liquid and gaseous contents of the bowels, the ptomains or poisons developed in them from fermentation, producing extremely depressing effects on the nervous system, with great derangement of the stomach and assimilative organs, as shown in the pale faces, debility, neuralgia, headache and a general feeling of exhaustion; but, we get, in addition, from impacted fæces in the rectum, uterine displacement, with its consequent disturbance in the pelvic circulation and with its general reflex neuroses. It is a well known fact to gynecologists that the left ovary is oftener diseased than the right one. The left ovarian vein has no valve and a slight pressure upon it prevents its emptying. Doubtless the pressure of a loaded rectum in this event is a prolific cause of disease in the ovaries, especially the left. It should be a part of the college curriculum to force girls to go to the closet immediately after breakfast. A careless retention of urine is frequently the cause of displacement of the uterus backward-thereby producing a prolapsis of the ovary, resulting in congestion and inflammation of it. Floating kidney is a common factor, often unrecognized, in producing invalidism in women. Many symptoms are produced by it similating diseases of the genital organs, and, unless very careful examination is made, the real cause of the trouble is overlooked and undetected and the poor woman fails to get the relief that she seeks at our hands. Great disturbance in the assimilating organs, as well as in the nervous system, are frequently traceable to this cause, and it is the duty of every physician in examining a woman in poor health, to make a thorough investigation as to the position of the kidney. Undoubtedly one of the greatest causes of ovarian and tubular disease in the girl or young woman is the exanthematous fevers, a cause entirely too much neglected and overlooked in attending girls with measles, small-pox, and especially scarlet fever, and I might add, mumps. Sufficient prominence has not been given to these. It is clearly our duty to explain to mothers the dangers of this disease to their daughters, that they may give us their co-operation in the cases that come under our care and observation, and especially for the protection of those not attended by physicians. Inflammation of the ovaries and tubes from this cause may be complicated, and often is by inflammation of the uterus and pelvic peritoneum, resulting in adhesions. Cirrhosis of the ovary frequently follows inflammation of it, occurring as a sequel to the exanthema. You are all familiar with the cirrhosis of the testicles following orchitis from mumps. Inflammation of the tubes causing hydrosalpinx, frequently resulting in permanent occlusions, is not as uncommon as is supposed after these fevers.

Early marriage is another great source of disease in women. I cannot better express my views than to quote from Garrigues, who says: "Puberty is the period when the possibility of reproduction begins, and by no means the time when it is desirable that the girl should marry and become a mother. Statistics show a very great mortality among married women under twenty years of age. It is evidently against nature's laws that women should become mothers before they are grown. Their uteri should have attained their maximum development, and their breasts should be developed for supplying nourishment for their infants. The pelvis should have reached a size that will allow the passage of a full-grown child, the muscles should have acquired strength to propel it, and the whole system should be endowed with the full power of resistance and endurance. It can, therefore, be said that women must reach twenty before this condition of affairs can, with safety, be assured, and therefore, should not marry under this age."

Marrying with pre-existing pelvic diseases should always be discouraged, until the disease is cured, or serious results are likely to follow, rendering the woman an invalid and not infrequently destroying the happiness of the home, as the husband feels that he is the victim of deception. All girls, where there is the least suspicion of pelvic disease, should be examined before marrying (not necessarily, indeed, very seldom, by the vagina) but by the rectal and abdominal palpation. If a diseased condition exists marriage should be deferred until this is cured. Furthermore, it is our duty to impress upon those found unsuited for the marriage relation, the importance of allowing us to make known their real condition to their "fiance" for their double protection, the preservation of their health and the protection of their honesty of purpose. If this course was insisted upon, many invalids would be saved, the poor women spared years of suffering and the happiness of many homes continued. If the tubes and ovaries are found tender and sensitive to the gentle touch of a well-trained finger, often producing intense pain, what must she expect and what will surely follow, if, in this condition, she enters the marriage state?

Childbirth and efforts to prevent conception are doing an injury to the women of America that cannot be computed. There are, perhaps, two causes for this. With the one, she cannot be deprived of the pleasures of society by the care of children and she would risk anything to her health rather than forego this satisfaction; and second, she is weak, nervous and worn down already, probably the victim of pelvic disease; has probably borne one or more children and feels in her wretched condition that she cannot endure the pain and suffering of childbirth; she has not the stamina to stand the strain. Surely this poor woman must excite our sympathy and most certainly would if we could realize what she suffers. Certainly the means used to prevent conception are frequently the direct cause of pelvic disease. Vaginal injections made immediately after coition, (when the blood vessels are distended and the nerves in the greatest state of excitement and tension, certainly a time when nature calls for rest) are frequently used, with water at a very low temperatnre, chilling the parts and producing congestion and inflammation, as shown by hemorrhages, leucorrhœa, etc. Chemicals and numerous mechanical devices are often employed, all of which produce the same result. Unfortunately it does not stop here, for, if conception occurs, the development of the embryo must, at all hazards, be arrested and the product gotten away at any cost short of death and this is sometimes the price paid to accomplish it. Every physician of intelligence must admit that the evils of childbirth may be, and they certainly are, less than those following criminal abortion. The serious effects of these on the future health of the woman, to say nothing of its moral aspect, is well known to every gynecologist. Exhaustive hemorrhage, retained membranes, metritis, uterine fungosities, slow sepsis producing ovarian and tubal disease, resulting frequently in occlusion of the tubes, with permanent sterility and in ovarian and tubal abscesses. These are some of the results following abortion. See the very small families of this age. This can only be accounted for, first, by the means used to prevent conception, and second, sterility from pelvic disease, or abortion. The accidents of childbirth are very, very often the cause of serious or even permanent illness in many instances. Many of these accidents, such as cervical and perineal tears are, in the hands of even the most skillful obstetricians, absolutely unavoidable, and it should always be so stated to the husband and wife. Quite recently I heard a venerable practitioner in the city of Philadelphia say: "In a large obstetrical practice, extending over a period of more than forty years, I have never had but one case where there was the slightest laceration." I felt very much inclined to tell him, which was certainly the case, that he did not have sense enough to recognize them when they occurred. Don't you know that his carelessness and want of knowledge has put thousands of dollars into the pockets of the Philadelphia gynecologists, to say nothing of the suffering, and perhaps loss of life, of the poor, innocent victims of his ignorance? I have tried to be careful in my obstetrical work, but have had them to occur in my practice; so has every man in my hearing who has the ability to discover and the honesty to acknowledge it. I regret to admit, but it is too true, that carelessness and ignorance in the physician is responsible for severe lacerations, in many cases unrecognized. Sometimes the busy or impatient physician, tired of waiting, or else anxious to make another call, to prevent his professional brother from entering his clientele, gives a few doses of harmless (?) ergot. What is the result? A quick release from his detention, a woman with a badly torn cervix or perineum, possibly unrecognized. This picture is not overdrawn; and here I must say, that while ergot has its place in obstetrical practice, and a valuable one it is, I honestly believe it has done more damage to women in the free and indiscriminate way in which it has been used, than all the remedies in the entire materia medica, with possibly one exception, opium; and it is a question in my mind whether or not it should be excepted, for it is undoubtedly true that many of our opium habitues are first the victims of ergot. The abused, too frequent and unskilled use of the forceps are often productive of great damage to the maternal soft tissues. While the unnecessary and unskilled use of the forceps should be condemned, yet they are certainly a boon to suffering women. When timely and properly used, they may not only save the mother from serious accident, which might result in chronic invalidism, or even loss of life, but they have saved the lives of many thousands of children. When lacerations do occur, they should at once be repaired under the strictest aseptic precautions. Every man who practices obstetrics should always examine his patient thoroughly after a labor, and even if a slight cervical or perineal tear exists, he should go prepared to close it at once. If he does not feel competent or is unwilling to do it, let him call for assistance. If done at once while the parts are so benumbed from pressure, an anæsthetic is rarely necessary; but, the greatest reason, however, for the immediate repair, is to close the avenue for septic infection. The abraded or torn surface is the gateway through which septic material passes to the pelvic organs and peritoneum, producing puerperal fever, so called, septic peritonitis, salpingitis and ovaritis, often resulting in an accumulation of pus in the peritoneum, ovaries and tubes. There is no more virulent disease, that the abdominal surgeon has to deal with, none that runs so rapid a course, as sepsis following childbirth. When it occurs, if the poor victim escapes with her life, she is perhaps an invalid for the balance of her days. She may be rendered sterile by occluded tubes, from inflammatory pressure, or, should she escape with a patulous tube and enough healthy ovarian tissue be left to again become pregnant, she may be so broken down physically as to render her unable to again assume the responsibilities of mother. Slight and unrecognized tears in cases of unclean obstetrics are much oftener the cause of serious illness of the mother than is generally believed. Here is the cause, in most instances, of childbed fever. Where women escape septic contamination, cervical and perineal tears lead frequently to a most disastrous train of reflex symptoms, as well as produce local disease such as sub-involution, with its long and disastrous train of nervous symptoms. I must not fail to call attention to the want of cleanliness on the part of some physicians when making vaginal and uterine examinations, as a potent factor in the causation of pelvic diseases. Not only should the hands and instruments be clean, but the vagina and cervix should be thoroughly cleansed and disinfected before an instrument of any kind is passed into the uterus. How many of you have seen your professional brother swab the uterus with all sorts of medicaments, using dirty cotton on an equally unclean applicator, never cleansing the vagina and cervix, and even passing a sound into the uterus that had been passed in perhaps a dozen others without coming in contact even with soap and water. While the uterine sound is a valuable instrument in its place, there are but few instances where it is required. I believe it has done more damage to women than all other instruments combined, and it not only has been, but is still, too indiscriminately used. Stem pessaries have made many victims for the surgeon's knife. With the possible exception of the uterine sound, they have done more harm than any other instrument or appliance.

The damage that has come from the above line of tinkering gynecology cannot be estimated.

My subject is so broad that I have been forced to omit even allusion to many points I desired to discuss. Many I have mentioned I have not been able, for fear of making this paper too long, to discuss as freely as their importance demanded. I shall crave your indulgence a little longer while I discuss briefly, the deadliest of all enemies to female health, gonorrhœa. Undoubtedly, many, yes, very many, of the chronic diseases of the ovaries and tubes that come under the observation of gynecologists are due to gonorrhœa.

In a majority of the cases the poor woman is entirely ignorant of the factthat she has, or has ever had, any specific disease. Indeed, her husband may tell you, examined or questioned, that months or even years before his marriage he was a subject of gonorrhœa, which was cured and has since shown no evidence of a return. Many of the highest authorities now seriously doubt whether gonorrhœa in the male is ever cured; that the subject may be apparently well for months or years, and that under certain excesses, notably venereal, it returns and the wife is promptly infected; indeed, she may be without any perceptible return of the urethritis in the husband. From such excesses as above mentioned, the poison or gonnococcus, which has been latent for so long, quietly resting in its bed, is aroused to fresh life and vigor to do its deadly work on the innocent and unsuspecting woman. As general practitioners, we are all too careless in the treatment of gonorrhœa in the male. We have regarded it as a disease, under ordinary circumstances, easily cured, and have discharged our patients as well and ready for marriage soon after the discharge and other local symptoms disappear. Mr. Tait says: "We know that a man never really gets cured of gonorrhœa. Under any stimulus of wine or women, it will come back and be effective. From the enormous number of cases of damaged uterine appendages that have come under my care in the young married woman, who has remained sterile, after having been a few months married, I am almost disposed to believe that it is unjustifiable for a man who has ever suffered from gonorrhœa to enter the marriage state at all." What a startling statement from so high an authority.

As to gonorrhœa in women. Most of us have been taught to regard it as a most trivial disease; nothing more than a vaginitis, easily cured by a few vaginal irrigations with some astringent solution. I well remember in my college days that a distinguished Philadelphia professor, now dead, taught us that equal parts of apple vinegar and cold water thrown into the vagina was a sure cure and absolute specific. I wrote down this prescription and in the beginning of my professional career, as my work was confined largely to the lower classes, where gonorrhœa was often found, God only knows how many women I left to be ultimately relieved only by the surgeon's knife. Doubtless many of my hearers, especially the older ones, have received the same teaching and have furnished their quota for the operating table. Happily, science has thrown much light upon this, formerly, a very dark field of our professional labor, and the man who now lightly treats gonorrhœa in the woman, is either ignorant of his professional duty, or is even worse, indifferent to the result. So great an authority as Lawson Tait, says: "Early in life I heard an eminent surgeon say that if he was doomed to have a venereal disease, he would rather have syphilis than gonorrhœa. I marveled and disbelieved, but now I know that if he included women in his thoughts of the subject, he spoke truly. Syphilis is relatively a harmless disease. It may and does cause discomfort, distress and even pain, but I doubt if it ever kills the woman. Even if it does, where syphilis kills ten, gonorrhœa kills its thousands, and it would take the suffering of one hundred cases of syphilis to make up for the long, weary years of agony of one case of gonorrhœa —pyo-salpinx."

Truly such a statement from one of such broad learning and extensive experience as Mr. Tait must impress us with the great importance of this subject. Sinclair says: "The surgeons, until of late years, have accepted it only as a trifling incident, and is clear that the obstetricians who had better opportunities for knowledge have entirely overlooked the fact. Modern gynecologists have unearthed the conclusion that gonorrhœa is a terrible and fatal scourge to women." Gonorrhœa in the female is not always easily diagnosed, and doubtless many hundreds of cases are passed, by competent and intelligent physicians, as a simple vaginitis. In many cases of vaginitis, it is often an impossibility to get a history of specific infection, and it is often unwise to press our questions too far. Many, in fact, most of the cases of acute gonorrhœa in women, go to the family physician for advice. They are ignorant of even the possibility of any specific trouble, for they well know no unfaithfulness to their husband has been practiced, nor do they suspect infidelity on his part. The family physician, knowing the character of the lady, and not fully realizing the possibility of redevelopment of the disease in the male, never suspects gonorrhœa, regards the trouble as trivial in its character and so treats it; when, if its real nature had been recognized and the disease treated accordingly, the future ill effects upon the poor woman might have been averted. Many cases of gonorrhœa occur where no vaginitis exists, at least, to an extent to make it noticeable; or, even when it is to a considerable extent, the gynecologist is rarely consulted until the uterus and its appendages are involved; perhaps months or years having passed before he sees the patient. She is then probably broken down in health, a great sufferer, perhaps bedridden, a confirmed invalid with great pus sacs in the fallopian tubes, or ovaries, or both, but it is rare to have pus in an ovary without also having it in a tube. While the acute symptoms of gonorrhœa in the woman usually subside without immediate trouble, still there are in my opinion, many cases of acute and even fatal peritonitis, where the cause has been and is clearly and undoubtedly due to the extension of acute gonorrhœal inflammation through the uterus and tubes. As already mentioned there may be entire absence of vaginitis, the virus affecting the uterus direct passing through it to the tubes and ovaries, giving us endometritis of the most disagreeable character.

The entire organ, after a while, will become affected by extension through the tubes, and pelvic peritonitis, resulting in adhesions of all the pelvic organs and causing an endless amount of suffering and pain, and finally in chronic invalidism. Inflammation of the tubes is soon followed by a restriction in their calibre, and is clearly shown by the terrible suffering occurring frequently twenty-four to forty-eight hours before the menstrual flow begins. Finally, the tube is entirely sealed at its uterine end and we have hopeless and incurable sterility. The poor woman soon becomes a confirmed invalid, looking to the return of each menstruation with dread and horror, for she well knows the pain and suffering that awaits her. Her digestion is deranged, her nutrition impaired and she becomes weak, exhausted and unable to stand or walk without the greatest suffering; the approach of her husband is productive of most intense pain, and in short, she never knows a moment of relief from her suffering. Such a picture, I am sure, should enlist the most determined effort on our part to arrest the spread and extension of the gonorrhœal virus. The changes in the uterine mucous membrane from gonorrhœa are often productive of abortions. Many of the cases of so-called puerperal fever are due to the rupture during labor of a pyo-salpinx, most likely of gonorrhœal origin; one tube has remained patulous and healthy, while the other was the seat of an undetected pus sac. Rupture of these produce septic peritonitis, so-called puerperal fever. Of course, the presence of pus in the tubes is much more dangerous to the woman during pregnancy and labor than at any other time. Many cases of ruptured pus tubes have resulted in death to the poor woman, when the cause has been overlooked and perhaps attributed to inflammation of the bowels, gastritis, etc. It has been my privilege, recently to examine many specimens of pus tubes due to gonorrhœal infection, and it has also been my pleasure as well, to see the victims taken almost from articulo-mortis by the surgeon's knife, the only remedy for these cases.

Now, gentlemen, I do not claim to have presented anything especially new; still if I have, in any way, roused the hard, honest and industrious workers in our profession to give more attention to the prevention of diseases in women, I have fulfilled my purpose.

